# Machine learning computational tools to assist the performance of systematic reviews: A mapping review

**DOI:** 10.1186/s12874-022-01805-4

**Published:** 2022-12-16

**Authors:** Ramon Cierco Jimenez, Teresa Lee, Nicolás Rosillo, Reynalda Cordova, Ian A Cree, Angel Gonzalez, Blanca Iciar Indave Ruiz

**Affiliations:** 1 International Agency for Research on Cancer (IARC/WHO), Evidence Synthesis and Classification Branch, Lyon, France; 2 Laboratori de Medicina Computacional, Unitat de Bioestadística, Facultat de Medicina, Universitat Autònoma de Barcelona, Bellaterra, Spain; 3 International Agency for Research on Cancer (IARC/WHO), Services to Science and Research Branch, Lyon, France; 4 Servicio de Medicina Preventiva, Hospital Universitario 12 de Octubre, Madrid, Spain; 5 International Agency for Research on Cancer (IARC/WHO), Nutrition and Metabolism Branch, Lyon, France; 6 Department of Nutritional Sciences, University of Vienna, Vienna, Austria

**Keywords:** Systematic reviews, Mapping review, Evidence-based practice, Software development, Machine learning, Automatization

## Abstract

**Background:**

Within evidence-based practice (EBP), systematic reviews (SR) are considered the highest level of evidence in that they summarize the best available research and describe the progress in a determined field. Due its methodology, SR require significant time and resources to be performed; they also require repetitive steps that may introduce biases and human errors. Machine learning (ML) algorithms therefore present a promising alternative and a potential game changer to speed up and automate the SR process. This review aims to map the current availability of computational tools that use ML techniques to assist in the performance of SR, and to support authors in the selection of the right software for the performance of evidence synthesis.

**Methods:**

The mapping review was based on comprehensive searches in electronic databases and software repositories to obtain relevant literature and records, followed by screening for eligibility based on titles, abstracts, and full text by two reviewers. The data extraction consisted of listing and extracting the name and basic characteristics of the included tools, for example a tool’s applicability to the various SR stages, pricing options, open-source availability, and type of software. These tools were classified and graphically represented to facilitate the description of our findings.

**Results:**

A total of 9653 studies and 585 records were obtained from the structured searches performed on selected bibliometric databases and software repositories respectively. After screening, a total of 119 descriptions from publications and records allowed us to identify 63 tools that assist the SR process using ML techniques.

**Conclusions:**

This review provides a high-quality map of currently available ML software to assist the performance of SR. ML algorithms are arguably one of the best techniques at present for the automation of SR. The most promising tools were easily accessible and included a high number of user-friendly features permitting the automation of SR and other kinds of evidence synthesis reviews.

**Supplementary Information:**

The online version contains supplementary material available at 10.1186/s12874-022-01805-4.

## Background

Evidence-based practice (EBP) establishes a rigorous approach to gathering and summarising the best available evidence within a specific field or research purpose [1–3]. This paradigm has significantly changed the discourses and practices in various fields such as biomedical sciences, education, medicine, psychology, and public policy [3–7]. Evidence-based medicine (EBM) developed these principles to identify and evaluate medical information and provide structured summaries of the available evidence to inform decision in health care and improve the diagnosis and treatment of patients [1, 3, 8]. Systematic reviews (SR) are evidence synthesis studies that follow a structured method and are considered the most reliable source of evidence in the hierarchy of levels of evidence [9–11]. A SR aims to select, identify, critically appraise, and synthesise the best available evidence within pre-specified eligibility criteria to answer a clearly defined research question [9, 12-14]. This practice allows the consolidation of large amounts of findings from publications and the identification of potential evidence gaps in a specific field. Without SR, decision-making processes are vulnerable to bias and would be often only based on a subset of studies that may not be representative of the knowledge base of the field. In addition, information overload due to increasing number of scientific publications, publication bias and heterogeneity of reporting, are challenges faced during decision-making process. These raise the risk of obtaining biased results and flawed conclusions, and accurate evidence synthesis are key in many fields to informing the decision-making process. Promoting, enhancing, and facilitating the production of SR is therefore vital in the use of the best available evidence to inform healthcare decision-making processes [15–17].

The number of published systematic reviews has increased exponentially in recent years [18, 19]. However, conducting a SR is still a complex, challenging and time-consuming process [20, 21], and it requires a multidisciplinary team with at least one experienced reviewer [22]. The use of computational tools to assist and facilitate various stages of conducting a SR has always been relevant and the development of new tools has also seen a progressive increase [23–25]. Currently many tools are available [23–26], some of them providing support during some stages of the SR process and others supporting the entire workflow [27–33]. The types of software used can vary; including algorithms, packages (collections of functions or algorithms), libraries (collections of packages), desktop apps (programs that are executable from the desktop), and they may range from being locally run from a device to web-based applications (software accessible and executable through a web browser) that are hosted on a webserver.

As *the WHO Classification on Tumours* Programme (WCT) [34, 35] we wish to promote evidence-based practice in pathology. We need to review a very large amount of scientific literature to classify each of the 3,000 tumour types in the classification, ideally applying structured evidence synthesis methods by conducting SR. To produce so many SR with limited human resources there is a need for computational assistance. The WCT and EBP in general would benefit considerably from computational assistance to perform SR [3, 36] but tools that adapt well to the particularities of the fields of pathology and cancer diagnosis are not available.

**Fig. 1:**
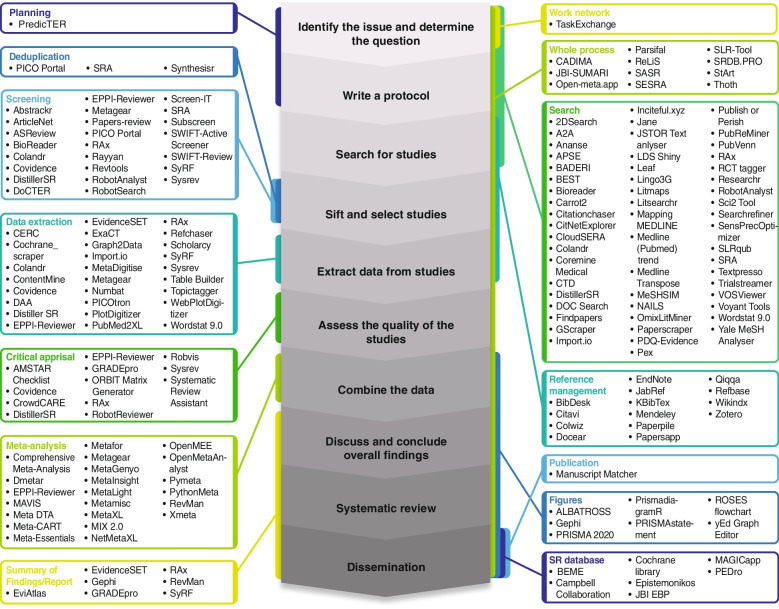
Software tools assisting the Systematic Review process. The central figure represents from the early to the late stages of the systematic review process described by the Cochrane Foundation (*Higgins JPT et al., Cochrane Handbook for Systematic Reviews of Interventions version 6.3, Cochrane, 2022*). The obtained tools were grouped into blocks depending on the covered SR step (Planning: Process of planning and writing the protocol for a SR; Deduplication: Process of removing duplicated records and articles retrieved by the search in a SR; Screening: Process of screening records and articles retrieved by the search in a SR; Data Extraction: Process of data extraction in the SR included studies; Critical appraisal and/or Bias assessment: Evaluation of the methodological quality and/or risk of bias in a SR included studies; Meta-analysis: Process of pooling findings of included studies, using statistical methods; Summary of findings/Report: Process of summarizing and reporting of findings; Work network: Process of networking; Whole process: whole systematic review process including all steps; Search: process of elaboration of search strategy, running the search and/or obtaining records retrieved by the search; Reference Management: process of screening and selection of records, as well reporting and scientific writing allowing management of large numbers of reference records and in text citations; Figures: Visualisation of data; SR Databases: Registration, collection and dissemination of SRs; Publication: Process of publishing the results)

The number of software tools and workflows to support the performance of systematic reviews, systematic maps, and meta-analyses is growing rapidly [29, 37–39]. Use of natural language processing (NLP) and machine learning (ML) algorithms to reduce time and workload in the SR process is becoming increasingly popular [29, 40, 41]. However, despite significant progress, integration of high-quality methodological approaches with user-friendly applications is rare. Well adapted open-source software is also rare, and integration among the different software tools is poor. A vast number of free and fee-based tools exist, but there is a lack of validation and consensus when it comes to identifying which tool best fits specific needs. This limits the utility of computational tools, being especially difficult to find solutions to assist specific steps of the SR. At present day some of the computational tools (e,g., web-applications, algorithms, executables, etc.) assisting the SR process are shown in Fig. 1, they can be found in online catalogues/repositories like the *SR toolbox* [42]. Around 160 tools assist the reviewer in either one specific step (during record search [43], screening [39, 44, 45], data extraction [45], risk of bias assessment/ critical appraisal, etc.), or guide the user through several steps or the whole SR process [46–48].

Especially promising are ML techniques [49–51] for the automation of systematic reviews steps [41, 46, 52]. Within the artificial intelligence (AI) discipline, ML methods are considered the most promising techniques for working with unstructured data. These methods, usually combined with NLP technologies are used for text classification and data extraction, result in effectively assisting the article screening process during the performance of SR [29, 53, 54]. Machine learning is a multidisciplinary field that consists of the development of computer algorithms that can “learn” how to perform a specific task [51]. By using mathematics and statistics, the algorithms are trained to make classifications or predictions based on a provided set of training data, driving decision-making within specific applications. Unlike conventional algorithms, ML systems pretend to imitate human learning behaviour and can improve their performance without being directly re-programmed [54, 55]. So far ML algorithms are primarily employed to assist the article screening during the systematic review process. This process of screening publication records implies categorising them into groups (i.e., included or excluded), considering the research question and predefined eligibility criteria. Article selection is usually performed by two independent human reviewers, revising first title and abstract of the retrieved records, and later full text of the article. The first step compromising the revision of title and abstract of a bibliometric record, is a task for which ML algorithms can be employed. These algorithms can be trained to develop the ability to categorise, using so called “training data sets” of records screened by human reviewers. This application of ML could be used to facilitate updating systematic reviews, since the categorisation from the original review can be used to train the algorithm for the screening of recently published records. These algorithms are trained according to the computer–human interaction where the availability of a training data set and the purpose of the classification strategy are the key points [41, 49, 51, 55]. They can be broadly classified as supervised learning (trained on labelled data), unsupervised learning (trained without labelled data) and semi-supervised learning (trained by a small, labelled dataset and a large unlabelled data set) algorithms [53].

As mentioned above, these algorithms/software are more and more used for article screening in systematic reviews [53, 55, 56], with some examples being the programs *Abstrack®* [39], *ASReview®* [56], *Colandr®* [33], EPPI-Reviewer*®* [57] and *Rayyan®* [33], all easily accessible online. Other steps of the SR process such as data extraction [54, 58] or risk of bias assessment [59] have been also been exploring whether ML tools can facilitate the work. As an example, the software *RobotReviewer® is able to* assign low, high or unclear risk of bias to randomized control trials (RCTs) [59]. Additionally, the development of automatic data extraction tools is being investigated (for instance *DistillerSR®* [41, 46]), and important efforts are underway to explore whether ML tools can be used efficiently in combination with each other.

Most steps in the SR process can potentially benefit from automation [60], but they often require more sophisticated computational methods than those provided by ML [44]. However, developing automatic screening tools based on combined ML techniques seems feasible; plenty of research and developments have been done in this field in recent years [26, 39, 41, 46, 52]. Considering these advances, the rapid evolution of the area, and the difficulties in identifying the best suited tool for each task and field, we aimed with this project to systematically map available ML tools that assist the SR process [61]. No other mapping review on this topic has been published, and our findings will identify existing tools, detect potential development gaps, and help to guide future research towards the most promising areas.

## Methods

We conducted a Mapping Review to identify existing ML tools to assist during the SR process. A protocol was registered in the *Open Science Forum* (OSF) platform [62] (Available at https://osf.io/wmy7n/?view_only=c501b501ede84b96b3c3353e3e81deb0), since *the International Prospective Register of Systematic Reviews (PROSPERO)* does currently not accept registrations for mapping reviews.

To identify all relevant ML tools comprehensive searches in several bibliographic databases and software repositories were performed. Additionally searching in software repositories allowed us to identify tools not mentioned in journal articles, conference abstracts, or similar technical literature.

### Search and selection

A tailored search strategy was developed in collaboration with an information specialist (TL) to search for relevant publications in the electronic databases MEDLINE (through *PubMed)*, *EMBASE* and *Web of Science*. Database specific terms (MeSH and Emtree) and keywords for the concepts of “*Systematic review*” and “*Machine learning (ML)*” were combined with Boolean operators to produce tailored search strings for each database. Multiple variations of search terms were combined to produce different sets of results. Final search strategies are available as supplementary material (See Additional file 1).

In addition, a structured search was conducted to identify ML tools in the following repositories of software: *The Comprehensive Perl Archive Network* (CPAN) [63], *The Comprehensive R Archive Network (CRAN)* [64], *GitHub *[65], *The National Centre for Text Mining (NaCTeM)* [66], *The PHP Extension Community Library (PECL)* [67], *The Python Package Index (PyPI)* [68], *SourceForge *[69] and the *Systematic Review Toolbox (SR toolbox)* [70]. Due to the limitations of the search engines of these websites no search strings could be used to retrieve records and multiple, iterative searches using single keywords were performed. Searches were conducted in the mentioned electronic databases and software repositories from 01^st^ of April to the 31^st^ of May 2021, with no language restrictions.

### Eligibility criteria

Any publication or repository record describing a ML software to assist the SR process in any field was considered eligible. All publications or records with sufficient technical description (reporting at least the software name, a short tool description and its purpose) were included when reporting in English, Spanish, German, or French, and when developed or updated within the last 10 years. Reference management software and tools that were not accessible to the reviewers (i.e., downloadable, importable, or executable from their source) were excluded.

Publication records retrieved by the search in electronic databases were imported into *EndNote®* and duplicates were removed. Records were screened for eligibility based on their titles and abstracts by two reviewers (RCJ and BII) independently. Full text PDFs were obtained for all abstracts deemed relevant for inclusion and further assessed against the inclusion criteria by the same two reviewers independently to obtain a final number of included publications. Discrepancies were resolved by consensus.

Records retrieved by the search in repositories were registered in an ad hoc developed data base using Microsoft Excel® and duplicates were removed. Records were screened for eligibility based on their titles and summaries provided by the first reviewer (RCJ). A second reviewer (BII) revised the selection, resolving disagreement by consensus.

### Data extraction and synthesis

A data extraction form was developed, piloted, and refined to capture basic information for each identified tool. Extracted data included: *computer science method* applied for the development of the tool (e.g., natural language processing, supervised learning), *stage of systematic review process the tool assists with*, tool *release date or publication date of the description*, *date of last update*, licensing and pricing details (free access or requiring payment for the complete version), as well as being *open-source (the source code is available and repurposed) or not*, and the *source to access/download the tool* (e.g., software repository, hyperlink). In addition, the retrieved tools were categorised according to how much background in programming is needed to use the software, considering mainly whether the tool implementation needs modifications that require advanced programming skills (e.g., creation or adaptation of algorithms to use functions within a package).

Extracted data were compiled in a summary of findings (SoF) table and identified tools graphically summarised in a mapping infographic.

## Results

**Fig. 2:**
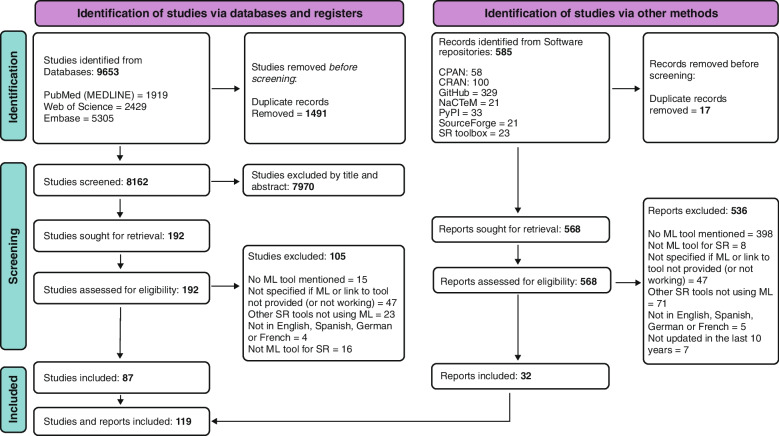
PRISMA flow diagram for the mapping review. *Adapted From:* Page MJ et al. 2021**.** SR: Systematic Review; ML: Machine Learning; CPAN: The Comprehensive Perl Archive Network; CRAN: The Comprehensive R Archive Network; NaCTeM: National Centre for Text Mining; PyPI: The Python Package Index

**Fig. 3:**
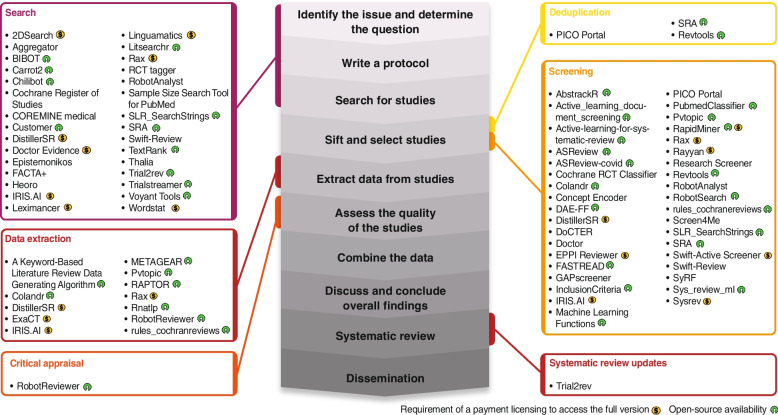
Machine learning tools that assist the Systematic Review process. The central figure represents from the early to the late stages of the systematic review process described by the Cochrane Foundation (Higgins JPT et al., Cochrane Handbook for Systematic Reviews of Interventions version 6.3, Cochrane, 2022). The obtained ML tools were grouped into blocks depending on the covered SR step. Next to the tool names, the yellow symbol means the requirement of a payment licencing to access to the full version of the tool and the green symbol open-source availability. SR = Systematic Review, ML = Machine learning

A total of 9653 studies were retrieved from the structured searches in bibliometric databases (*PubMed*, *Embase* and *Web of Science*), 1491 of which were duplicates. Of the remaining 8162 items that were screened, 7970 were excluded during a review of title and abstract (see Fig. 2). The remaining 192 publications were assessed by full text, resulting in 105 studies being excluded for reasons as not reporting on a ML tool [15], not providing a link to the tool [47], not using ML methods [23], using ML but not being adapted for SR [16], or not being published in previously defined languages [4]. 86 publications were identified describing ML tools and included. In addition, a total of 585 records were obtained from the selected software repositories (*CPAN*, *CRAN*, *GitHub*, *NaCTeM*, *PyPI*, *SourceForge* and *SR toolbox*), 17 of which were duplicates. The remaining 568 records were screened, excluding 536 and selecting 32 that described ML tools. After the screening process, a total of 119 descriptions of ML tools from publications and repository records were included for the data extraction process (See Fig. 2). From those descriptions 63 tools that assist the SR process using ML techniques were identified (Table 1). We described basic information for each of the identified ML tools, including the step of the SR where it operates, pricing options, open-source availability, computational methods involved, number of citations/times mentioned in this review, date of the last update, hyperlink to the tool, and the necessity of a programming background to use the software in Table 1. To facilitate the tools description in the table they were grouped by category of application (e.g., algorithms, web-applications). In addition, we graphically represented the identified ML tools into blocks depending on the covered SR step (see Fig. 3).

**Table 1:** Identified machine learning tools that assist the systematic review process by type of computational tool

Index	ML tool name	Step in the systematic review process the tool is used	Computational methods involved	Last update	Number of citations	Hyperlink to access to the tool
Machine learning algorithms						
1	A Keyword-Based Literature Review Data Generating Algorithm (*)	Data extraction	NLP	2019	1	http://www.mdpi.com/2073-8994/12/6/903/s1
2	Active_learning_document_screening (*)	Screening	NLP, SL	2020	1	https://github.com/afcarvallo/active_learning_document_screening
3	Active-learning-for-systematic-review (*)	Screening	NLP, SL	2019	1	https://github.com/sxzhang1201/active-learning-for-systematic-review
4	DAE-FF (*)	Screening	SL	2019	1	https://github.com/gkontonatsios/DAE-FF
5	InclusionCriteria (*)	Screening	NLP	2019	1	https://github.com/infoqualitylab/InclusionCriteria
6	Machine Learning Functions (*)	Screening	SL	2020	1	https://systematicreviewsjournal.biomedcentral.com/articles/10.1186/s13643-020-01520-5#Sec14
7	PubmedClassifier (*)	Screening	NLP, SL	2018	1	https://github.com/YujiaBao/PubmedClassifier
8	RAPTOR (*)	Data extraction	NLP	2021	1	https://github.com/CochraneSchizophrenia/RAPTOR
9	Rules_cochranereviews (*)	Screening, Data extraction	NLP	2020	1	https://github.com/dsurian/rules_cochranereviews
10	SLR_SearchStrings (*)	Search, Screening	NLP	2018	1	http://bit.ly/2PwL37v
11	TextRank (*)	Search	NLP, USL	2020	2	https://cran.r-project.org/web/packages/textrank/index.html
Desktop-applications						
12	ASReview	Screening	NLP, SL	2021	2	https://asreview.nl
13	ASReview-covid	Screening	NLP, SL	2021	1	https://github.com/asreview/asreview-covid19
14	FASTREAD (*)	Screening	SL	2020	1	https://github.com/fastread/src/tree/v1.4.0
15	GAPscreener	Screening	NLP, SL	2017	2	http://hugenavigator.net/HuGENavigator/HNDescription/opensource_GAP.html
16	Pvtopic (*)	Screening, Data extraction	SL	2016	1	http://nactem.ac.uk/pvtopic
17	RapidMiner	Screening	NLP, SL, SSL, USL	2021	6	http://rapid-i.com
18	Rax	Search, Screening, Data extraction	NLP, SL	2021	1	https://raxter.io
19	SWIFT-Review	Search, Screening	SL	2019	8	https://www.sciome.com/swift-review
Software packages/libraries						
20	BIBOT (*)	Search	NLP	2018	1	https://github.com/Nurtal/BIBOT-light-version
21	Costumer (*)	Search	SL	2017	2	https://github.com/UBESP-DCTV/costumer
22	Litsearchr (*)	Search	NLP	2019	2	https://github.com/elizagrames/litsearchr
23	Revtools (*)	Deduplication, Screening	NLP	2019	2	https://cran.rproject.org/package=revtools
24	Rnatlp (*)	Data extraction	NLP	2020	1	https://github.com/SensorNet-UFAL/rnatlp
Web-applications						
25	2DSearch	Search	NLP	2021	2	https://www.2dsearch.com
26	Abstrackr	Screening	SL	2019	23	http://abstrackr.cebm.brown.edu
27	Aggregator	Search	SL	2017	1	http://arrowsmith.psych.uic.edu/cgi-bin/arrowsmith_uic/RCT_Tagger.cgi?ID=22379
28	Carrot2	Search	NLP	2021	1	https://search.carrot2.org
29	Chilibot	Search	NLP	2017	1	http://www.chilibot.net
30	Cochrane RCT Classifier	Screening	SL	2021	1	https://crsweb.cochrane.org/login.html
31	Cochrane Register of Studies	Search	NLP	2021	1	https://community.cochrane.org/help/tools-and-software/crs-cochrane-register-studies
32	Colandr	Screening, Data extraction	NLP, SL	2018	6	www.colandrapp.com
33	Concept Encoder	Screening	NLP, SL	2021	1	https://www.fronteo.com/en/products/conceptencoder
34	COREMINE medical	Search	NLP	2021	1	http://www.coremine.com/medical
35	DistillerSR	Search, Screening, Data extraction	NLP, SL	2021	10	https://www.evidencepartners.com
36	DoCTER	Screening	NLP, SSL	2017	1	https://www.icf-docter.com
37	Doctor Evidence	Search	NLP	2021	3	https://www.drevidence.com
38	Epistemonikos	Search	SL	2021	3	https://www.epistemonikos.org
39	EPPI-Reviewer	Screening	NLP	2021	17	https://eppi.ioe.ac.uk/eppireviewer-web
40	ExaCT	Data extraction	SL	2010	7	https://bio-nlp.org/EXACT
41	FACTA +	Search	NLP	2019	1	http://www.nactem.ac.uk/facta
42	Heoro	Search	NLP	2021	1	https://www.heoro.com
43	IRIS.AI	Search, Screening, Data extraction	NLP, SL	2019	1	https://the.iris.ai
44	Leximancer	Search	NLP, USL	2021	2	https://www.leximancer.com
45	Linguamatics	Search	NLP	2021	1	https://www.linguamatics.com
46	PICO Portal	Screening, Deduplication	NLP, SSL	2021	1	https://picoportal.org
47	Rayyan	Screening	NLP, SL	2021	17	https://www.rayyan.ai
48	RCT tagger	Search	NLP, SL	2017	6	http://arrowsmith.psych.uic.edu/cgi-bin/arrowsmith_uic/RCT_Tagger.cgi
49	Research Screener	Screening	NLP, SL	2021	1	https://researchscreener.com
50	RobotAnalyst	Search, Screening	SL	2018	11	http://www.nactem.ac.uk/robotanalyst
51	RobotReviewer (*)	Data extraction, Risk of Bias	NLP, SL	2020	14	https://www.robotreviewer.net/
52	RobotSearch	Screening	NLP, SL	2019	5	https://github.com/ijmarshall/robotsearch
53	Sample Size Search (SSS) Tool for PubMed	Search	NLP	2020	1	https://ihealth.uemc.es
54	Screen4Me	Screening	NLP	2020	1	https://community.cochrane.org/sites/default/files/uploads/S4M_webinar_slides_Feb_2019.pdf
55	SRA	Search, Deduplication, Screening	NLP	2021	5	https://sr-accelerator.com
56	SWIFT-Active Screener	Screening	SL	2021	4	https://www.sciome.com/swift-activescreener
57	SyRF	Screening	NLP	2021	2	http://syrf.org.uk
58	Sysrev	Screening	SL	2021	1	https://sysrev.com
59	Thalia (*)	Search	NLP	2019	2	http://nactem-copious.man.ac.uk/Thalia
60	Trial2rev (*)	Search, SR updates	SSL	2021	1	https://github.com/evidence-surveillance/trial2rev
61	Trialstreamer	Search	NLP, SL	2021	1	https://trialstreamer.robotreviewer.net
62	Voyant Tools	Search	NLP	2021	1	https://voyant-tools.org
63	Wordstat	Search	NLP	2021	1	https://provalisresearch.com/products/content-analysis-software

*NLP*: Natural language processing; *SL*: Supervised learning; *SSL*: Semi-supervised learning; *USL*: Unsupervised learning. (*) = requirement of a programming background for the correct deployment of the tool

Among the 63 tools identified, the majority were cited between 1 and 5 times (52, 83%) within the screening of the 119 descriptions obtained from publications and repository records. The remaining tools (11, 18%) obtained between 6 and 23 citations, being *Abstrackr®* [23], *EPPI-Reviewer®* [57], *Rayyan®* [33], *RobotReviewer®* [14], and *RobotAnalyst®* [11] the top 5 most referenced tools in this review.

All the identified software tools assist early stages of the SR process, with the 63 (100%) tools addressing automation of the steps of literature search, screening, and data extraction; 30 (47%), 35 (55%) and 11 (17%) tools respectively. Whereas late SR stages have been less well-exploited, with 1 tool count.

In terms of updates over time most identified tools have been updated or released over the last 5 years (62, 98%), whereas only 1 (2%) case was considered nearly out of date due to older updates (see Table 1). It is worth mentioning that around half of the identified tools have been released or updated over the last year (29, 46%).

Thirty-nine (62%) of the included tools were web applications, 11 (17%) were algorithms, 5 (8%) were libraries or packages and 8 (13%) were desktop applications. Also, 22 (35%) tools required a programming background to be used whereas 41 (65%) could be used by a less skilled customer (see Table 1).

The most applied AI methodology by the tools was NLP 48 (76%), mostly to improve, accelerate, or automate the underlying text analytics. Supervised learning was employed in more than half of the tools 37 (58%) whereas, unsupervised and semi-supervised algorithms were less frequently utilized.

In terms of open-source status, 31 (49%) of the retrieved tools were open-source, 15 (24%) were not accessible at all and for 17 (27%) the accessibility was not described. Most of the identified tools were freely available (44, 70%), but 14 (22%) required payment for a license and in 5 (8%) cases this was not clearly described.

When comparing licencing requirement with the updates over time we observed that within the 14 fee-based tools, only 1 (13%) were outdated, while 13 (92%) have been updated over the last five years and 13 (86%) in the last year. In contrast, from the 44 freely available tools, 1 (15%) remained out to date, 43 (97%) were updated over the last five years and 13 (30%) were updated in the last year.

We also detected that of the 44 free tools, 30 (68%) were open-source, 5 (12%) were not, and 9 (20%) were not described. In contrast, from the 14 fee-based tools, 10 (72%) were not open-source, one (7%) was open-source and three (7%) were not described.

Variations in tools’ software considering open-source availability and licencing were also noticeable. On one hand, from the 39 identified web applications, only 10 (26%) were open-source, while 14 (36%) were not open-source, and 15 (38%) were not described. In contrast, of the 11 algorithms all were open-source. The 5 libraries and packages had similar an equal profile, where all were open-source. Amongst the 8 desktop apps, 5 (63%) were open-source, 1 (12%) was not open-source, and in 2 (25%) cases this was not described. Algorithms and packages/libraries had the highest proportion of freely available tools with 5 (100%) and 11 (100%) respectively, compared to web and desktop applications, with 23 (59%) and 5 (63%) tools respectively.

## Discussion

SR and meta-analyses are recognized as the highest level of evidence [3, 9, 11, 13, 17], and the growing number of available tools to assist during the performance of SR probably reflects an increasing recognition of the utility of this type of studies (see Fig. 1). It probably also reflects an increasing appreciation of the potential value of computational methods to simplify the performance of such highly structured reviews of the scientific literature, and also to improve their reliability and reproducibility. Recent years have seen the development of many ML tools that aim to reduce the immense human resources and time effort required by a multi-disciplinary team to develop such a review [29, 38, 52, 56]. One of the most assisted steps in the whole review process is the article screening, where these tools assist the reviewer by suggesting, classifying, or selecting records, and can either help or even replace the reviewer during certain parts of the process [28, 52, 71]. However, the current ML algorithms require evaluation and training using a pre-selected and labelled set of records, and their performance varies greatly depending on this previous step of training [41, 71, 72]. These requirements have hampered extended use of such tools by systematic reviewers for a long time and constitute an important barrier for their use in in topics with no representative training data sets to train the algorithms, as is often the case in the field of pathology. Also, when reviewing the retrieved tools more closely, it is obvious that most are not adapted to be used by users with no background in informatics or programming skills, who would require a tool with a user-friendly interface. Despite recent advances, increasing numbers of developments and new computational solutions to assist during the SR process, it remains a challenge for a reviewer to select the best suited software for each SR project. The results of this mapping review provide an overview of the currently available ML tools to assist during the performance of a SR and will help future reviewers and researchers to identify the right tool for each project and facilitate the development of new evidence synthesis methodologies (see Fig. 3).

It is evident that the ML tools identified by this review have been created with different aims by a great variety of developers, ranging from individuals and small research groups to large organisations dedicated to evidence-based medicine and systematic reviewing at a large scale [31, 37, 44, 56, 57]. These efforts have shown widely variable success so far and our final tool map shows that only a few tools are suitable for use by reviewers without programming backgrounds. Nevertheless the high proportion of free (70%) and open-source (49%) tools we have detected in this mapping review may indicate efforts by the SR community to overcome these limitations and produce tools to facilitate systematic review production for all types of users. We believe that there are signs of a growing movement of developers in the field that will probably continue to promote the progress of the automation of SR steps. It is crucial that such emerging collaborations continue to be facilitated in the future and open sharing of data as well as methods being promoted [23, 25, 26, 29]. Interestingly, amongst the free tools about 68% are open-source, showing once more that the two concepts of open-source and free-of-charge are similar, but describe views based on fundamentally different values: open-source is a methodology used to facilitate the development of software in a given field/task, while free software is a social movement aiming to provide equity in access. We identified also 14 (22%) ML tools that require the payment for a licence, and as expected, 10 (72%) are not openly accessible. This may point to an interest of private developers in these types of tools and their potential commercial value [57, 73, 74]. The access to such privately developed ML tools will be limited to the organisations or individuals that can afford their fees and will therefore not be an option for all reviewers. However, some of the payment-based tools might provide free access or fee reduction purchase depending on the review purpose and/or the team conditions (e,g., collaborating memberships, shared interests), as applying substantial discounts to lower middle-income country (LMIC) users and other similar situations.

Most of the identified tools (98%) were updated or released during the last 5 years, and 48% of all retrieved ML tools during the last year, which proves an increasing interest in ML software development in the field and probably indicates a marked demand for such tools. Not surprisingly, ML tools requiring payment for a license were far more frequently updated during the last year (86%) than freely accessible tools (30%), showing the advantage of the licenced approach for the rapid development of ML tools. However, most free tools (97%) have also been released or updated during the last 5 years, suggesting that this approach can also be efficient and produce updated products without relying on commercial strategies. Some not-for-profit research institutions appear to be highly interested in the development and promotion of SR automatization tools that are made freely available, which may in part be compensating for the funding disadvantage [26, 75].

Our findings show a major interest of developers in computational methods to assist the early stages of the SR process specifically during the screening of articles. It is in this step where automation seems to have greater potential for success, with 55% of the tools assisting in this step and being the most promising of ML solutions. The other two SR steps with promising developments are literature searching and data extraction, with 47% and 17% of the tools respectively. This seems to point towards an existing interest in the improvement of those stages using ML approaches, but probably less success in the development. Within the other SR stages, a reduced number of tools was identified, describing a minor interest or lack of potential for ML solutions for these SR stages.

It is understandable that web-applications are the biggest group of retrieved ML tools (62% of all tools, being 59% of them freely available and 26% open-source). This type of software generally permits easy access directly from a web browser and a user-friendly interface that doesn’t require any advanced knowledge of the software or programming background for a successful use. Utility and acceptability are likely to be high for these types of tools due to intuitive interfaces and potential to adapt to different reviewer profiles, but we have not been able to assess this in our review due to a lack of reporting of such features. However, web-based applications require extensive resources and a host (be it an institution, group, or enterprise) to provide web server maintenance, technical support, and to warrant a proper implementation of the tool. These resources are not always available for developers and it this is likely the reason for the large number of libraries/packages and algorithms we retrieved. These constitute the second largest block comprising 25% of all retrieved ML tools and are fully developed, but inactive software tools that require further steps for their implementation. These tools comprise one or more algorithms and require knowledge in informatics and programming skills for their application, which significantly reduces the tools’ usability. Hence not a solution for all reviewers, but still a valuable tool that permits customization and can be tailored to the specific needs of a project with the necessary skills. Interestingly, ML packages/libraries and algorithms showed the highest proportion of free (100% both) and open-source (100% both) tools compared to the other types of software described. Desktop applications were rather rare with 8 mapped ML tools, even though this type of tool allows the user to locally execute the software from a computer after installation. This process and laborious implementation steps may limit their usability, but no programming skills are required, and this may turn them into one of the most promising solutions. This may be even more so the case if an easy installation and compatibility with common operating software can be assured. However, our findings suggest that further developments are needed and that at this stage it results still difficult to assess which type of software is best suited for single review projects and reviewer profiles. Factors such as research topic, composition and expertise of the review team, available resources, and technical skills, still need to be considered and are the challenges for future development.

Only a few tools obtained more than 5 citations either in scientific publications or software repositories. Not surprisingly, among those were some of the best-known and most used tools as *Abstrackr®* [39], EPPI-Reviewer*®* [57], *Rayyan®* [33], and *RobotReviewer®* [31] (see Table 1).However, the majority of identified tools (83%) were cited less than 5 times, suggesting that despite the increasing development of software to assist the SR process, new tools will have to compete with a few dominant well-known tools. Efforts to improve the diffusion of the newly developed tools is therefore key, together with an improvement of currently applied methodology.

We believe that collaboration to improve already available ML tools may yield well adapted software that can provide a wide range of functionalities needed for systematic reviews, as shown by the already existing variety of ML tools and the recent acceleration in the launch of new and updated version. There are projects such as *Metaverse* [76], where developers collaborate to collect, integrate, and expand available functions, following open-source principles and making the tools freely accessible to the evidence synthesis community. Other projects as *SR-Accelerator* [28], integrate several tools in a suite to assist in more than one step of the SR procedure, aiming to produce software that guides and assists the reviewer during the whole process. Additionally software repositories or toolboxes such as the *SR toolbox* [42, 70] exist to promote and share already available tools that assist the SR process. Databases or repositories with specific training sets are also a resource that helps the community of developers perform collaborative work, providing the necessary platforms for the sharing of data, information, and expertise.

Following this successful development, more efforts should be undertaken to facilitate communication and knowledge exchange among developers and users, so that usability and functionality of already existing tools can be improved and adapted to the needs of different systematic review projects. Training in SR automation for reviewers, provision of basic programming skills, and plain language explanations on how to adapt tools to specific needs, may also speed up the development of better ML tools, or even promote the creation of new ones.

Our systematic mapping review holds potential for bias inherent to the limitations of its methodology. However being a mapping exercise, risk of bias as that of selective reporting [77], could be minimized by applying few exclusion criteria and reporting on all identified tools for which we could retrieve sufficient information. This also avoided a potential selection bias, and by following a previously defined and registered protocol we assured the reliability and reproducibility of our work. The lack of advanced search functionalities in the search engines of software repositories did not permit sophisticated search strategies and ML tool registries might not have been detected. Nevertheless, the iterative search process in these repositories combined with the sensitive search strategy applied in the bibliographic databases strengthen the completeness of our findings, and the high number of records screened makes this mapping review highly reliable. However, due to fast evolution of the targeted field, new potential tools have been developed since the performance of this project, being the Elicit tool [78] an example of a tool that the developed search strategy haven’t considered. Despite the fact that the applied methodology does not provide a synthesis of the findings or a critical appraisal of the methodological quality of the retrieved publications, our mapping exercise has value and pertinence. Our description of available tools, visually summarized in two comprehensive infographics provide a decision support tool for reviewers, researchers and other decision-makers conducting and funding evidence synthesis projects. This mapping review covers the breadth of science in ML tools and is needed to assist related questions. The unique overview that it provides will inform future reviewers, developers, and research in the field.

## Conclusion

Systematic reviews (SR) are considered the most reliable source in the hierarchy of the evidence levels, they permit the combination of large amounts of findings from scientific publications and the identification of potential evidence gaps in a field. Without SR, decision-making processes are exposed to bias and flawed conclusions. The development of computational tools to assist the systematic review process is rapidly expanding, this reflects an increasing interest on the production of this type of studies. Our review provides an overview of available software to assist the performance of SR according to SR steps, and a complete map of ML tools, showing that ML algorithms represent one of the most investigated methods for the assistance of SR. The most promising approaches focus on the automation or semi-automation of parts of the process and include a high number of easy to use and easy to access web-based applications that permit the use of ML software for SR and other kind of evidence synthesis reviews. Our results have uncovered the current state of open-source development and how it could support a call for the formation of collaborative working groups in this field. Promoting and facilitating the production of SR by using computational assistance is therefore crucial in the use of the best available evidence to inform healthcare or any decision-making processes.

## Supplementary Information

**Additional file 1: Supplementary file1.** Search strategies used in the mapping review. The file contains three tables with the developed search strategies in the electronic databases MEDLINE, EMBASE and Web of Science.

## Data Availability

All data generated or analysed during this study are included in this published article and its additional files.

## Abbreviations

SR: Systematic Review
EBP: Evidence-Based Practice
EBM: Evidence-Based Medicine
ML: Machine Learning
AI: Artificial Intelligence
NLP: Natural Language Processing
SL: Supervised Learning
SSL: Semi-Supervised Learning
USL: Unsupervised Learning
