# Supplementary material for: Machine learning computational tools to assist the performance of systematic reviews: A mapping review
Source: BMC Med Res Methodol. 2022 Dec 16;22:322. doi: 10.1186/s12874-022-01805-4 (PMC9756658; doi:10.1186/s12874-022-01805-4)
Supplement: Supplementary file 1 — Additional file 1: Supplementary file1. Search strategies used in the mapping review. The file contains three tables with the developed search strategies in the electronic databases MEDLINE, EMBASE and Web of Science. [file 12874_2022_1805_MOESM1_ESM.docx]

**Supplementary Table 1. Search strategy performed in PubMed.**

| **Pubmed** | |
| --- | --- |
| **Database specific terms (MeSH)** | |
| **Index** | **Search strategy** |
| 1 | Review Literature as Topic[MH] |
| 2 | Systematic Reviews as Topic[MH] |
| 3 | Meta-Analysis as Topic[MH] |
| 4 | Artificial Intelligence[MH] |
| 5 | Data Mining[MH] |
| 6 | Information Storage and Retrieval /methods[MH] |
| 7 | Pattern Recognition, Automated[MH] |
| **Keywords** | |
| **Index** | **Search strategy** |
| 10 | meta-review*[tw] OR metareview*[tw] OR rapid review*[tw] OR scoping review*[tw] OR umbrella review*[tw] OR evidence review*[tw] OR literature review*[tw] OR systematic review*[tw] metaanalys*[tw] OR meta-analys*[tw] |
| 11 | active learning[tw] OR adaptive algorithm*[tw] OR artificial general intelligence[tw] OR AGI[tw] OR artificial intelligence[tw] OR artificial narrow intelligence[tw] OR ANI[tw] OR automated learning[tw] OR automatic classification[tw] OR automatic learning[tw] OR automatic term recognition[tw] OR backward chaining[tw] OR clustering engine*[tw] OR clustering tool*[tw] OR cognitive computing[tw] OR computational intelligence[tw] OR computational linguistic*[tw] OR computer reasoning[tw] OR computer vision system*[tw] OR connectionist model*[tw] OR data crunching[tw] OR data mining[tw] OR datamining[tw] OR deep learning[tw] OR document cluster*[tw] OR entity annotation*[tw] OR entity extraction*[tw] OR file cluster*[tw] OR forward chaining[tw] OR general AI[tw] OR generative adversarial network*[tw] OR GAN[tw] OR GANs[tw] OR hierarchical learning[tw] OR hyperparameter*[tw] OR knowledge discovery[tw] OR knowledge engineering[tw] OR learning algorithm[tw] OR learning scenario*[tw] OR linguistic analy*[tw] OR linguistic annotation*[tw] OR literature mining[tw] OR machine intelligence[tw] OR machine learning[tw] OR machine perception[tw] OR machine translation*[tw] OR natural language generation[tw] OR NLG[tw] OR natural language processing[tw] OR NLP[tw] OR natural language understanding[tw] OR NLU[tw] OR neural network*[tw] OR ANN[tw] OR ANNs[tw] OR RNN[tw] OR RNNs[tw] OR overfitting[tw] OR pattern recognition[tw] OR perceptron[tw] OR predictive analytics[tw] OR query expansion[tw] OR reinforcement learning[tw] OR semisupervised learning[tw] OR semisupervised model*[tw] OR sentiment analyses[tw] OR sentiment analysis[tw] OR strong AI[tw] OR structured prediction[tw] OR supervised learning[tw] OR supervised model*[tw] OR support vector machine[tw] OR SVM[tw] OR SVMs[tw] OR text cluster*[tw] OR text engineering[tw] OR text mining[tw] OR textmining[tw] OR topic modelling[tw] OR transfer learning[tw] OR Turing test*[tw] OR unsupervised learning[tw] OR unsupervised model*[tw] OR weak AI[tw] |
| 12 | Diseases Category[Majr] |
| 13 | Abstrackr[tw] OR BibDesk[tw] OR Bioreader[tw] OR CADIMA[tw] OR Carrot2[tw] OR CitNetExplorer[tw] OR Colandr[tw] OR Coremine[tw] OR covidence[tw] OR CrowdCARE[tw] OR Data Abstraction Assistant[tw] OR Distiller SR[tw] OR DistillerSR[tw] OR Doctor Evidence[tw] OR EPPI-Reviewer[tw] OR EPPIReviewer[tw] OR EviAtlas[tw] OR GAPScreener[tw] OR GRADEpro[tw] OR GRADEproGDT[tw] OR JBI-SUMARI[tw] OR Journal/Author Name Estimator[tw] OR Leximancer[tw] OR LibSVM[tw] OR MAVIS[tw] OR Medical Text Indexer[tw] OR Meta-Analysis via Shiny[tw] OR Meta-CART[tw] OR Meta-Essentials[tw] OR MetaGenyo[tw] OR MetaInsight[tw] OR MetaLight[tw] OR MetaMap[tw] OR metamisc[tw] OR metaphor[tw] OR MetaXL[tw] OR NaCTeM[tw] OR NetMetaXL[tw] OR PDQ-Evidence[tw] OR PDQEvidence[tw] OR Parsifal[tw] OR Pimiento[tw] OR pubreminer[tw] OR RapidMiner[tw] OR Quetzal[tw] OR RCT tagger[tw] OR Rayyan[tw] OR ReVis[tw] OR revtools[tw] OR RobotAnalyst[tw] OR RobotReviewer[tw] OR RobotSearch[tw] OR robvis[tw] OR SWIFT-Active Screener[tw] OR SWIFT-Review[tw] OR SWIFTReview[tw] OR Sci2[tw] OR SensPrecOptimizer[tw] OR SyRF[tw] OR sysrev[tw] OR Table Builder[tw] OR TaskExchange[tw] OR TerMine[tw] OR Textpresso[tw] OR Thalia[tw] OR VOSViewer[tw] OR Voyant[tw] OR WordStat[tw] OR yEd Graph Editor[tw] |
| 14 | automat*[tw] OR bag of words[tw] OR crowd-sourc* [tw] OR crowdsourc*[tw] OR decision support[tw] OR in-the-loop[tw] OR keyword-based[tw] OR micro task*[tw] OR microtask* OR ontolog*[tw] OR PICO[tw] OR precision[tw] OR recall[tw] OR time saving*[tw] OR timesaving*[tw] OR work-load*[tw] OR workload*[tw] OR work-flow*[tw] OR workflow*[tw] OR semiautomat*[tw] OR speed up[tw] OR terminolog*[tw] OR ((article*[tiab] OR citation*[tiab] OR concept*[tiab] OR document*[tiab] OR evidence[tiab] OR reference*[tiab] OR snippet*[tiab]) AND (analys*[tiab] OR classif*[tiab] OR cluster*[tiab] OR evaluat*[tiab] OR extract*[tiab] OR filter*[tiab] OR frequenc*[tiab] OR identif*[tiab] OR prioritiz*[tiab] OR recogni*[tiab] OR relevance[tiab] OR retriev*[tiab] OR review*[tiab] OR screen*[tiab] OR select*[tiab] OR sift*[tiab] OR summar*[tiab] OR synthesi*[tiab])) |
| 15 | address[pt] or autobiography[pt] or bibliography[pt] or biography[pt] or case reports[pt] or comment[pt] or consensus development conference[pt] or consensus development conference, nih[pt] or dictionary[pt] or directory[pt] or duplicate publication[pt]or editorial[pt] or historical article[pt] or interactive tutorial[pt] or interview[pt] or lecture[pt] or legal case[pt] or legislation[pt] or letter[pt] or news[pt] or newspaper article[pt] or overall[pt] or patient education handout[pt] or periodical index[pt] or portrait[pt] or video-audio media[pt] or webcast[pt] |
| 16 | case report*[ti] OR case series[ti] |
| **Combined concepts** | |
| **Index** | **Search strategy** |
| 17 | #1 OR #2 OR #3 |
| 18 | #4 OR #5 OR #6 OR #7 |
| 19 | #17 OR #10 |
| 20 | #18 OR #11 |
| 21 | #19 OR #20 |
| 22 | #21 NOT #12 |
| 23 | #21 AND (#13 OR #14) |
| 24 | #22 OR #23 |
| 25 | #15 OR #16 |
| 26 | #24 NOT #25 |

**Supplementary Table 2. Search strategy performed in Embase.**

| **Embase** | |
| --- | --- |
| **Database specific terms (Emtree)** | |
| **Index** | **Search strategy** |
| 1 | "systematic review(topic)"/de |
| 2 | "meta analysis (topic)"/de |
| 3 | "artificial Intelligence"/exp |
| 4 | "machine learning"/exp |
| 5 | "data classification"/exp |
| 6 | "data clustering"/exp |
| 7 | "information retrieval"/exp |
| 8 | "natural language processing"/exp |
| 9 | "diseases"/exp/mj |
| 10 | book/de |
| 11 | erratum/de |
| 12 | editorial/de |
| 13 | letter/de |
| 14 | note/de |
| 15 | "case control study"/exp |
| 16 | "case report"/de |
| 17 | "prospective study"/de |
| 18 | "retrospective study"/de |
| **Keywords** | |
| **Index** | **Search strategy** |
| 19 | ('meta-review*' OR 'metareview*' OR 'rapid review*' OR 'scoping review*' OR 'umbrella review*' OR 'evidence review*' OR 'literature review*' OR 'systematic review*' OR metaanalys* OR 'meta-analys*'):ab,ti |
| 20 | (‘active learning’ OR ‘adaptive algorithm*’ OR ‘artificial general intelligence’ OR ‘AGI’ OR ‘artificial intelligence’ OR ‘artificial narrow intelligence’ OR ‘ANI’ OR ‘automated learning’ OR ‘automatic classification’ OR ‘automatic learning’ OR ‘automatic term recognition’ OR ‘backward chaining’ OR ‘clustering engine*’ OR ‘clustering tool*’ OR ‘cognitive computing’ OR ‘computational intelligence’ OR ‘computational linguistic*’ OR ‘computer reasoning’ OR ‘computer vision system*’ OR ‘connectionist model*’ OR ‘data crunching’ OR ‘data mining’ OR ‘datamining’ OR ‘deep learning’ OR ‘document cluster*’ OR ‘entity annotation*’ OR ‘entity extraction*’ OR ‘file cluster*’ OR ‘forward chaining’ OR ‘general AI’ OR ‘generative adversarial network*’ OR ‘GAN’ OR ‘GANs’ OR ‘hierarchical learning’ OR ‘hyperparameter*’ OR ‘knowledge discovery’ OR ‘knowledge engineering’ OR ‘learning algorithm’ OR ‘learning scenario*’ OR ‘linguistic analy*’ OR ‘linguistic annotation*’ OR ‘literature mining’ OR ‘machine intelligence’ OR ‘machine learning’ OR ‘machine perception’ OR ‘machine translation*’ OR ‘natural language generation’ OR ‘NLG’ OR ‘natural language processing’ OR ‘NLP’ OR ‘natural language understanding’ OR ‘NLU’ OR ‘neural network*’ OR ‘ANN’ OR ‘ANNs’ OR ‘RNN’ OR ‘RNNs’ OR ‘overfitting’ OR ‘pattern recognition’ OR ‘perceptron’ OR ‘predictive analytics’ OR ‘query expansion’ OR ‘reinforcement learning’ OR ‘semisupervised learning’ OR ‘semisupervised model*’ OR ‘sentiment analyses’ OR ‘sentiment analysis’ OR ‘strong AI’ OR ‘structured prediction’ OR ‘supervised learning’ OR ‘supervised model*’ OR ‘support vector machine’ OR ‘SVM’ OR ‘SVMs’ OR ‘text cluster*’ OR ‘text engineering’ OR ‘text mining’ OR ‘textmining’ OR ‘topic modelling’ OR ‘transfer learning’ OR ‘Turing test*’ OR ‘unsupervised learning’ OR ‘unsupervised model*’ OR ‘weak AI’):ab,ti |
| 21 | (‘Abstrackr’ OR ‘BibDesk’ OR ‘Bioreader’ OR ‘CADIMA’ OR ‘Carrot2’ OR ‘CitNetExplorer’ OR ‘Colandr’ OR ‘Coremine’ OR ‘covidence’ OR ‘CrowdCARE’ OR ‘Data Abstraction Assistant’ OR ‘Distiller SR’ OR ‘DistillerSR’ OR ‘Doctor Evidence’ OR ‘EPPI-Reviewer’ OR ‘EPPIReviewer’ OR ‘EviAtlas’ OR ‘GAPScreener’ OR ‘GRADEpro’ OR ‘GRADEproGDT’ OR ‘JBI-SUMARI’ OR ‘Journal/Author Name Estimator’ OR ‘Leximancer’ OR ‘LibSVM’ OR ‘MAVIS’ OR ‘Medical Text Indexer’ OR ‘Meta-Analysis via Shiny’ OR ‘Meta-CART’ OR ‘Meta-Essentials’ OR ‘MetaGenyo’ OR ‘MetaInsight’ OR ‘MetaLight’ OR ‘MetaMap’ OR ‘metamisc’ OR ‘metaphor’ OR ‘MetaXL’ OR ‘NaCTeM’ OR ‘NetMetaXL’ OR ‘PDQ-Evidence’ OR ‘PDQEvidence’ OR ‘Parsifal’ OR ‘Pimiento’ OR ‘pubreminer’ OR ‘RapidMiner’ OR ‘Quetzal’ OR ‘RCT tagger’ OR ‘Rayyan’ OR ‘ReVis’ OR ‘revtools’ OR ‘RobotAnalyst’ OR ‘RobotReviewer’ OR ‘RobotSearch’ OR ‘robvis’ OR ‘SWIFT-Active Screener’ OR ‘SWIFT-Review’ OR ‘SWIFTReview’ OR ‘Sci2’ OR ‘SensPrecOptimizer’ OR ‘SyRF’ OR ‘sysrev’ OR ‘Table Builder’ OR ‘TaskExchange’ OR ‘TerMine’ OR ‘Textpresso’ OR ‘Thalia’ OR ‘VOSViewer’ OR ‘Voyant’ OR ‘WordStat’ OR ‘yEd Graph Editor’):ab,ti |
| 22 | (‘automat*’ OR ‘bag of words’ OR ‘crowd-sourc*’ OR ‘crowdsourc*’ OR ‘decision support’ OR ‘in-the-loop’ OR ‘keyword-based’ OR ‘micro task*’ OR ‘microtask*’ OR ‘ontolog*’ OR ‘PICO’ OR ‘precision’ OR ‘recall’ OR ‘time saving*’ OR ‘timesaving*’ OR ‘work-load*’ OR ‘workload*’ OR ‘work-flow*’ OR ‘workflow*’ OR ‘semiautomat*’ OR ‘speed up’ OR ‘terminolog*’):ab,ti |
| 23 | (article* OR citation* OR concept* OR document* OR evidence OR reference* OR snippet*) near/2 (analys* OR classif* OR cluster* OR evaluat* OR extract* OR filter* OR frequenc* OR identif* OR prioritiz* OR recogni* OR relevance OR retriev* OR review* OR screen* OR select* OR sift* OR summar* OR synthesi*) |
| 24 | ‘case report*’:ti OR ‘case series’:ti |
| **Combined concepts** | |
| **Index** | **Search strategy** |
| 25 | #1 OR #2 |
| 26 | #3 OR #4 OR #5 OR #6 OR #7 OR #8 |
| 27 | #10 OR #11 OR #12 OR #13 OR #14 OR #15 OR #16 OR #17 OR #18 |
| 28 | #25 OR #19 |
| 29 | #26 OR #20 |
| 30 | #28 AND #29 |
| 31 | #30 NOT #9 |
| 32 | #30 AND (#21 #22 #23) |
| 33 | #31 OR #32 |
| 34 | #33 NOT (#27 OR #24) |

**Supplementary Table 3. Search strategy performed in Web of Science (WoS).**

| **Web of Science** | |
| --- | --- |
| **Database specific terms (Topic)** | |
| **Index** | **Search strategy** |
| 1 | TS=(metaanalys?s* OR metareview*) OR TS=((meta OR rapid OR scoping OR umbrella OR evidence OR literature OR systematic) near/1 (review*)) |
| 2 | TS=(“active learning” OR “adaptive algorithm*” OR “artificial general intelligence” OR AGI OR “artificial intelligence” OR “artificial narrow intelligence” OR ANI OR “automat* learning” OR “automat* classification” OR “automat* term recognition” OR “backward chaining” OR “clustering engine*” OR “clustering tool*” OR “cognitive computing” OR “computational intelligence” OR “computational linguistic*” OR “computer reasoning” OR “computer vision system*” OR “connectionist model*” OR “data crunching” OR “data mining” OR “datamining” OR “deep learning” OR “document cluster*” OR “entity annotation*” OR “entity extraction*” OR “file cluster*” OR “forward chaining” OR “general AI” OR “generative adversarial network*” OR GAN OR GANs OR “hierarchical learning” OR “hyperparameter*” OR “knowledge discovery” OR “knowledge engineering” OR “learning algorithm” OR “learning scenario*” OR “linguistic analy*” OR “linguistic annotation*” OR “literature mining” OR “machine intelligence” OR “machine learning” OR “machine perception” OR “machine translation*” OR “natural language generation” OR NLG OR “natural language processing” OR NLP OR “natural language understanding” OR NLU OR “neural network*” OR ANN OR ANNs OR RNN OR RNNs OR overfitting OR “pattern recognition” OR perceptron OR “predictive analytics” OR “query expansion” OR “reinforcement learning” OR “semisupervised learning” OR “semisupervised model*” OR “sentiment analys?s” OR “strong AI” OR “structured prediction” OR “supervised learning” OR “supervised model*” OR “support vector machine” OR SV OR SVMs OR “text cluster*” OR “text engineering” OR “text mining” OR “textmining” OR “topic modelling” OR “transfer learning” OR “Turing test*” OR “unsupervised learning” OR “unsupervised model*” OR “weak AI”) |
| 3 | TS=(Abstrackr OR BibDesk OR Bioreader OR CADIMA OR Carrot2 OR CitNetExplorer OR Colandr OR Coremine OR covidence OR CrowdCARE OR “Data Abstraction Assistant” OR “Distiller SR” OR DistillerSR OR “Doctor Evidence” OR “EPPI Reviewer” OR EPPIReviewer OR EviAtlas OR GAPScreener OR GRADEpro OR GRADEproGDT OR “JBI-SUMARI” OR “Journal/Author Name Estimator“ OR Leximancer OR LibSVM OR MAVIS OR “Medical Text Indexer” OR “Meta-Analysis via Shiny” OR “Meta-CART” OR “Meta-Essentials” OR MetaGenyo OR MetaInsight OR MetaLight OR MetaMap OR metamisc OR metaphor OR MetaXL OR NaCTeM OR NetMetaXL OR “PDQ-Evidence” OR PDQEvidence OR Parsifal OR Pimiento OR pubreminer OR RapidMiner OR Quetzal OR “RCT tagger” OR Rayyan OR ReVis OR revtools OR RobotAnalyst OR RobotReviewer OR RobotSearch OR robvis OR “SWIFT-Active Screener” OR “SWIFT-Review” OR SWIFTReview OR Sci2 OR SensPrecOptimizer OR SyRF OR sysrev OR “Table Builder” OR TaskExchange OR TerMine OR Textpresso OR Thalia OR VOSViewer OR Voyant OR WordStat OR “yEd Graph Editor”) |
| 4 | TS=(automat* OR “bag of words” OR “crowd-sourc*“ OR crowdsourc* OR “decision support” OR “in-the-loop” OR “keyword-based” OR “micro task*” OR microtask* OR ontolog* OR PICO OR precision OR recall OR “time saving*” OR timesaving* OR “work-load*” OR workload* OR “work-flow*” OR workflow* OR semiautomat* OR “speed up” OR terminolog*) OR TI=((article* OR citation* OR concept* OR document* OR evidence OR reference* OR snippet*) near/2 (analys* OR classif* OR cluster* OR evaluat* OR extract* OR filter* OR frequenc* OR identif* OR prioritiz* OR recogni* OR relevance OR retriev* OR review* OR screen* OR select* OR sift* OR summar* OR synthesi*)) OR AB=((article* OR citation* OR concept* OR document* OR evidence OR reference* OR snippet*) near/2 (analys* OR classif* OR cluster* OR evaluat* OR extract* OR filter* OR frequenc* OR identif* OR prioritiz* OR recogni* OR relevance OR retriev* OR review* OR screen* OR select* OR sift* OR summar* OR synthesi*)) |
| **Combined concepts** | |
| **Index** | **Search strategy** |
| 5 | #1 AND #2 AND (#3 OR #4) |
